# Incidence of upper respiratory tract infections with biological therapies in moderate to severe atopic dermatitis: a systematic review and meta-analysis

**DOI:** 10.3389/fmed.2025.1550640

**Published:** 2025-04-02

**Authors:** Rose Alraddadi, Mulham Kalantan, Yara Aljefri, Hadeel Maaddawi, Abdulrahman Alsamadani, Athoub Kadasa, Abdulrahman Softah, Baraa Tabbakh, Rahaf Alturkistani, Abdulhadi Jfri

**Affiliations:** ^1^College of Medicine, King Saud Bin Abdulaziz University for Health Sciences, Jeddah, Saudi Arabia; ^2^King Abdullah International Medical Research Center, Jeddah, Saudi Arabia; ^3^Department of Dermatology, College of Medicine, King Saud University, Riyadh, Saudi Arabia; ^4^Division of Dermatology, Department of Medicine, Ministry of the National Guard-Health Affairs, Jeddah, Saudi Arabia

**Keywords:** atopic dermatitis, biologics, dupilumab, IL4/13 inhibitor, tralokinumab, lebrikizumab, IL13 inhibitors, abrocitinib

## Abstract

**Introduction:**

Atopic dermatitis (AD) is a chronic inflammatory skin condition affecting 5%−20% of children and 2%−10% of adults worldwide. Treatment for moderate-to-severe AD includes biologics like dupilumab, tralokinumab, lebrikizumab, and JAK inhibitors (abrocitinib, upadacitinib). However, upper respiratory tract infections (URTIs) are commonly reported adverse events for these therapies. This meta-analysis aims to estimate the pooled incidence of URTIs associated with these treatments compared to topicals.

**Methods:**

A systematic search was conducted across PubMed, MEDLINE, DOAJ, and ClinicalTrials.gov for randomized controlled trials (RCTs) involving AD patients treated with dupilumab, tralokinumab, lebrikizumab, abrocitinib, or upadacitinib, excluding studies of patients treated with topicals, Studies on other dermatitis types and biologics. Data on URTI events, sample sizes, and incidence were extracted. Study quality was assessed using the Cochrane Risk of Bias Tool (RoB 2). A random-effects meta-analysis was conducted using the Netmeta package in R, calculating odds ratios (ORs) with 95% confidence intervals (CIs).

**Results:**

From 413 retrieved records, 21 studies met the inclusion criteria. URTI incidence of the treatment group in the included studies ranged from 0.35% to 41.5%, while control groups showed rates between 0% and 40%. Across all studies, URTI incidence was 9.70% in intervention groups and 8.03% in placebo groups (MH OR = 1.18, 95% CI: 0.98–1.42). Heterogeneity was low (*I*^2^ = 20.14%), with no evidence of publication bias (*p* = 0.83). There were no significant subgroup differences between patients taking different biological therapies (*Q* = 3.90, *p* = 0.42).

**Conclusion:**

While URTIs are common adverse events for AD therapies, their incidence in intervention groups is similar to control, suggesting no significant increase in risk. These findings provide critical insights for clinicians in balancing efficacy and safety when selecting therapies for AD patients. Further research should explore patient-specific risk factors for URTIs.

**Systematic review registration:**

Prospero registration code: [392093]. PROSPERO, Centre for Reviews and Dissemination: CRD42023392093.

## 1 Introduction

Atopic dermatitis (AD) is a chronic inflammatory skin disease with significant patient and population burden (1) affecting 5%−20% of children and 2%−10% of adults worldwide. AD is characterized by clinical signs of redness, swelling, excoriation, lichenification, and often, oozing/weeping and xerosis (2, 3). AD is thought to be a multifactorial disease that arises due to both genetic and environmental factors, although the complete pathophysiology has yet to be elucidated (4). For management of moderate-to-severe cases of AD, phototherapy and systemic immunosuppressants can be used (5). Dupilumab (anti-IL4/13) is one biologic that has been approved for AD, and more recently, tralokinumb (anti-IL13), lebrikizumab (anti-IL4/13), abrocitinib and upadacitinib [Janus kinases (JAKs) inhibitors], have been added (6, 7). Nemolizumab which target IL-31 has shown potential in reducing pruritus, although its overall efficacy in achieving EASI-75 responses is similar to placebo (8).

However, biologics targeting IL-22, IL-33, OX40, and thymic stromal lymphopoietin (TSLP) are in various stages of development, with some showing potential in early trials (9, 10). The development of biologics is moving toward a more personalized approach, aiming to address the unique immune profiles of different AD subsets (11).

While biologics present a promising treatment option for AD, several challenges persist. Limited long-term safety data, particularly in pediatric populations, raise concerns about their potential impact on an immature immune system (11). Additionally, their efficacy varies among patients (8), and the heterogeneous nature of AD—characterized by different phenotypes—necessitates a more personalized approach to treatment (11).

Additionally, concerns regarding adverse effects, particularly the risk of infections, have emerged as key considerations in evaluating these therapies. Upper respiratory tract infections (URTIs) and respiratory symptoms were one of the most frequently reported adverse events associated with dupilumab and the other approved biologics from clinical trials and real-world experience. Prior studies indicate that the incidence of URTIs in dupilumab-treated groups is generally similar to that in placebo groups. It is associated with a reduced risk of serious infections and non-herpetic skin infections, although it may slightly increase the risk of herpesviral infections (12–15). However, Tralokinumab showed URTIs as the most frequent treatment-emergent adverse event. Still, the incidence was similar between the treatment and placebo groups, suggesting no significant increase in risk (16). At the same time, JAK Inhibitors (Upadacitinib and Abrocitinib) have shown a higher incidence of URTIs than other therapies, with upadacitinib having a notably higher risk (17). We aimed to provide pooled incidence estimates using meta-analysis for the incidence of any URTI with dupilumab and other new agents, namely, lebrikizumab, tralokinumab, abrocitinib, and upadacitinib compared to topical.

## 2 Materials and methods

### 2.1 Search strategy

We systematically searched PubMed, MEDLINE, DOAJ, and ClinicalTrials.gov with no restrictions on language and the last date of searching data for studies assessing the incidence of any URTI with dupilumab and other new agents, namely, lebrikizumab, tralokinumab, abrocitinib, and upadacitinib in the treatment of AD in pediatric or adult populations. The search strategy was “[(Atopic dermatitis) OR (Atopic Eczema) AND (Dupilumab OR dupixent OR lebrikizumab OR Tralokinomab OR Upadacitinib OR Abrocitinib OR IL-4 inhibitors OR IL-13 inhibitors OR Janus Kinase 1 antagonist OR JAK1 inhibitor)].” A sample of the search strategy is detailed (Supplementary Table 1).

### 2.2 Eligibility criteria

The inclusion criteria of this systematic review were randomized controlled trials (RCTs) of adults and pediatrics diagnosed with AD and treated with JAK1 selective inhibitors (Updacitinib, Abrocitinib), or anti-IL4/13 (Dupilumab), or anti-IL13 (Tralokizumab, Lebrikizumab), excluding studies of patients treated with topicals, non-randomized clinical trials, Studies on other dermatitis types and biologics other than Dupilumab (OR) Updacitinib (OR) Abrocitinib (OR) Tralokizumab (OR) Lebrikizumab.

### 2.3 Data extraction

Two reviewers independently conducted data extraction and methodology quality assessment for all included studies. We extracted the type of treatment, the sample size for the treatment group, the sample size for the control group, and the incidence of URTI in the treatment group and the control group. For all studies, the main measure of interest was the incidence of URTI adverse events following the administration of Updacitinib, Abrocitinib, Dupilumab, Tralokizumab, and Lebrikizumab.

### 2.4 Quality assessment and risk of bias

Using the Cochrane Risk of Bias Tool (RoB 2), the studies were assessed across seven domains: random sequence generation, allocation concealment, blinding of participants and personnel, blinding of outcome assessment, incomplete outcome data, selective reporting, and other potential sources of bias (Supplementary Table 2).

### 2.5 Data management and software tools

The meta-analysis was conducted using the Netmeta statistical package in R. We extracted events and sample sizes for binary outcomes and mean (SD) and sample sizes for continuous outcomes. Odds ratios for binary outcomes and Standardized mean differences for continuous outcomes were calculated. Before NMA, we explored assumptions of transitivity among the studies by using several criteria, including tests for within-designs and between-designs inconsistency. We used a random effects model with the restricted maximum-likelihood estimator for tau^2^ to account for between-study variability. Odd ratios and 95% confidence intervals (CIs) were calculated to assess the effect sizes. Heterogeneity was assessed using the *I*^2^ and Cochran's Q statistic and subgroup analyses were performed to evaluate differences across drugs.

## 3 Results

**Figure 1:**
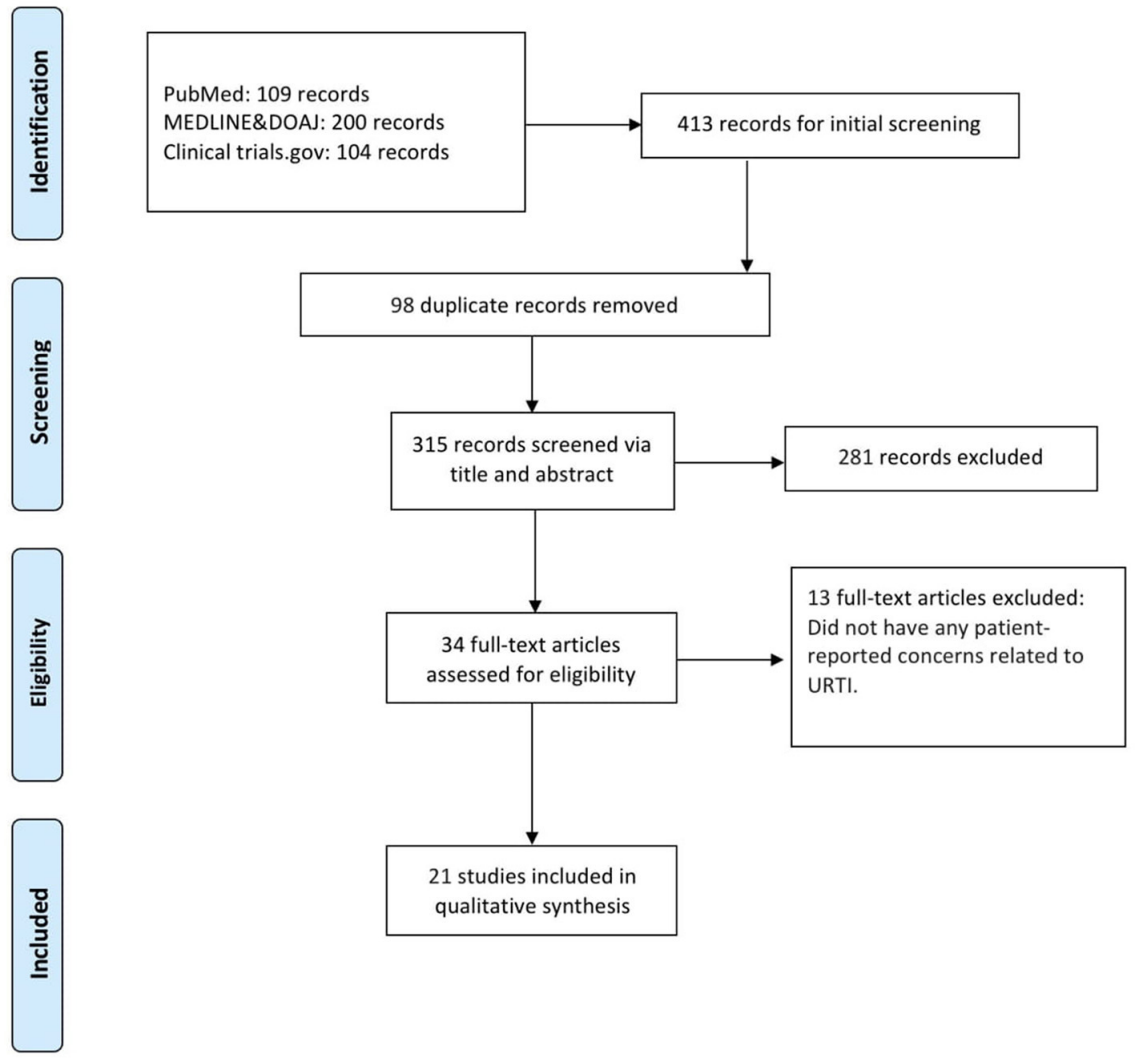
Study flowchart per the preferred reporting items for systematic reviews and meta-analyses (PRISMA) criteria.

We retrieved 413 records from various databases, including 109 from PubMed, 200 from MEDLINE & DOAJ, and 104 from ClinicalTrials.gov. After removing 98 duplicate records, a total of 315 records were screened based on their title and abstracts. Of these, 281 records were excluded due to irrelevance to the research focus. Subsequently, 34 full-text articles were assessed for eligibility. Among these, 13 articles were excluded as they did not address patient-reported concerns related to URTI. Ultimately, 21 studies were included in the qualitative synthesis (Figure 1).

Across 21 studies, the incidence of URTI in treatment groups varied widely, with rates as low as 0.35% (26) and as high as 41.5% (27). In control groups, the URTI incidence ranged from 0% (29) to 40% (27). In the study Wollenberg et al. (33), the treatment group had a higher URTI incidence than the control group (23.1% vs. 20.9%). Similarly, Simpson (18), the treatment group had a higher URTI rate than the control group (8.1% vs. 1.8%). Conversely, in studies like Simpson (30), the treatment group showed a slightly higher incidence (19.4%) than the control group (17.6%) (Supplementary Table 3).

**Figure 2:**
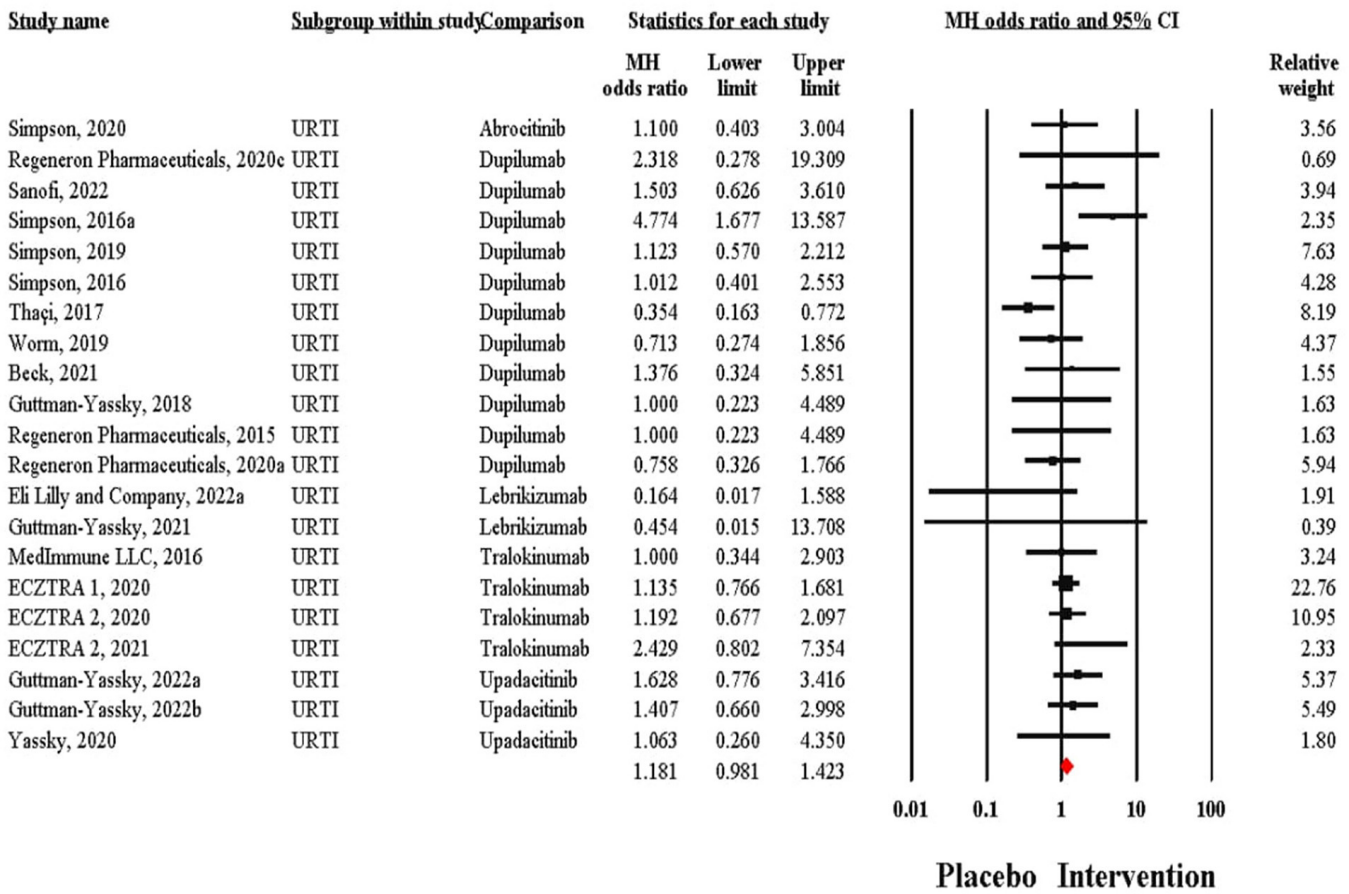
Forest plot presenting pooled effect size incidence of URTI adverse event with biological therapies in atopic dermatitis.

**Figure 3:**
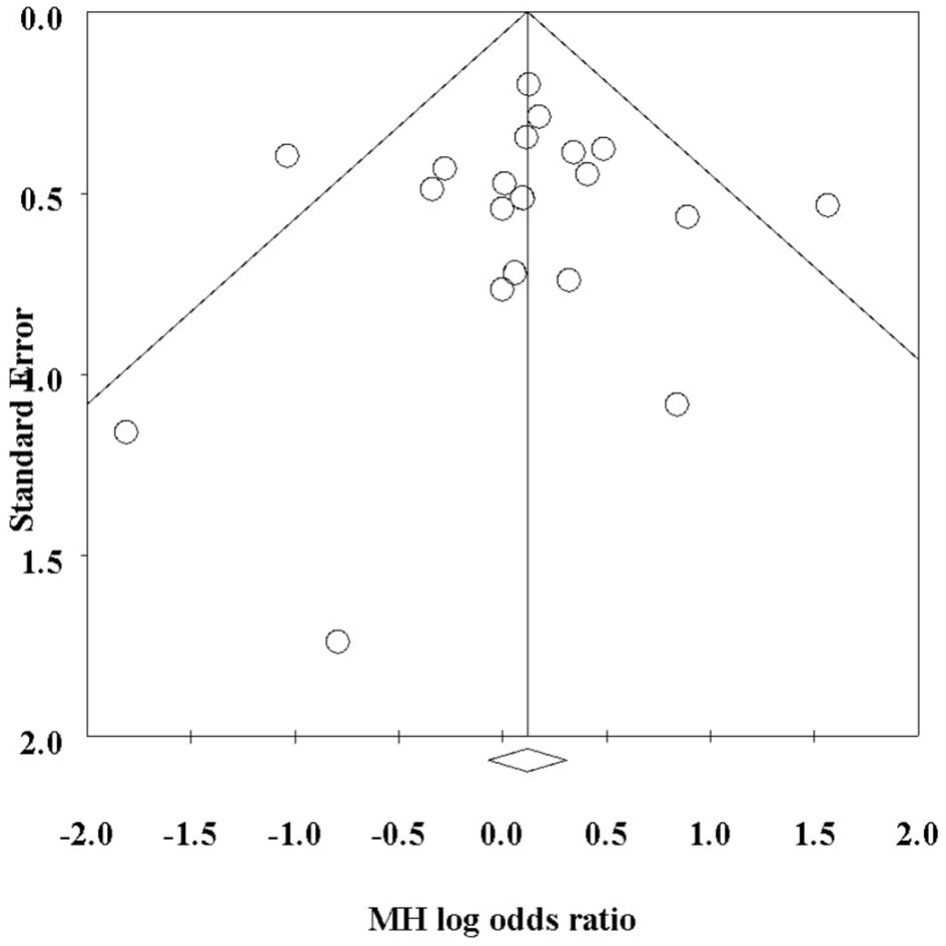
Funnel plot presenting evidence of minimal publication bias.

**Figure 4:**
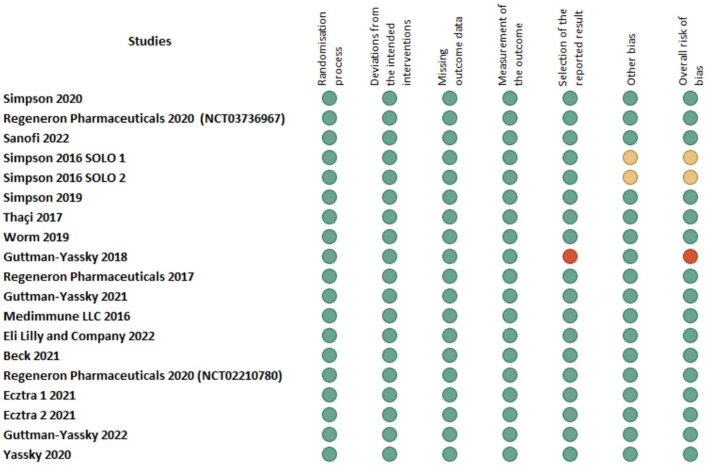
Risk of bias assessment for the included studies using the cochrane risk of bias tool (RoB 2).

Out of these 21 trials, 11 tested Dupilumab, 4 tested Tralokinumab, 3 tested Upadacitinib, 2 tested Lebrikizumab and one study tested Abrocitinib. Regarding the odds ratios for the incidence of URTI between control and intervention groups, in the Simpson (31) study, the odds ratio for Abrocitinib was 1.10 (95% CI: 0.403–3.004). Several studies using Dupilumab reported odds ratios ranging from 0.354 [95% CI: 0.163–0.772; (32)] to 4.774 [95% CI: 1.677–13.587; (18)]. For Lebrikizumab, odds ratios were as low as 0.164 [95% CI: 0.017–1.588; (16)] and up to 0.454 (95% CI: 0.015–13.708). Tralokinumab studies showed a range of odds ratios from 1.000 [95% CI: 0.344–2.903; (29)] to 2.429 [95% CI: 0.802–7.354; (33)]. Upadacitinib had odds ratios between 1.063 [95% CI: 0.260–4.350; (33)] and 1.628 [95% CI: 0.776–3.416; (33)]. There was minimal heterogeneity across the studies reporting this outcome (*Q* = 25.04, *I*^2^= 20.14%). Out of 5,053 intervention recipients, 490 (9.70%) reported URTI, compared to 182 (8.03%) control recipients. This translated to an MH Odds ratio of 1.18 (95% CI: 0.98–1.42) (Figure 2). There was no evidence of publication bias (*p* = 0.83) (Figure 3). There were no significant subgroup differences between patients taking different biological therapies (*Q* = 3.90, *p* = 0.42). The Risk of Bias assessment using ROB-2 for 19 studies revealed predominantly low risk across most domains, with a mix of low to high risk in the selection of the reported result and one study (28) showing high overall risk (Figure 4).

## 4 Discussion

This review aimed to explore the incidence of upper respiratory tract infection in patients with AD treated with different biological therapies. The overall incidence of URTI varied widely across the included studies, ranging from 0.35% to 41.5% among treatment groups compared to 0%−40% among controls. The biological agents used in those individual studies include Dupilumab, Tralokinumab, Upadacitinib, Lebrikizumab and Abrocitinib (19). There were no significant subgroup differences –regarding URTI incidence—between patients taking different biological therapies.

Dupilumab, an IgG4 monoclonal antibody, was the first biological agent described for treating AD. It improved pruritus symptoms and depression and anxiety symptoms. In addition to improving the overall quality of life (18–20).

The effectiveness of biological agents in the setting of AD was established in the literature; a review article demonstrated that many biological agents (Lebrikizumab, tralokinumab, fezakinumab, and nemolizumab) helped decrease disease severity. However, the safety profile of these agents needs to be established. Nevertheless, its use in pediatrics atopic dermatitis is to be investigated (21).

URTI was also reported in psoriasis patients treated with biological agents, as they are immunosuppressive (22). Other side effects of biological agents include conjunctivitis, especially with agents like dupilumab (19). Nemolizumab is currently under investigation. Trials at different stages showed good results in moderate and severe AD with good safety in the long term (10). However, the overall safety profile of these biological agents was said to be favorable, with side effects being mild to moderate (23).

Nevertheless, different biological agents used in the treatment of juvenile idiopathic arthritis were also associated with development of infections (URTI, pneumonia, and pleural effusion). In Rheumatoid arthritis patients treated with biological agents, higher rates of infections and hospitalization were reported especially with infliximab. However, apart from the biological agents which decrease the immunity, there are already patient's risk factors of infections like being immunocompromised, on steroid therapy and other factors that made them already susceptible to opportunistic infections (24, 25).

## 5 Limitations

Updated studies must be included in the analysis, as more evidence might have emerged. Also, more subgroup analysis can be done to differentiate between the sole effect of biological agents vs. the baseline patient's characteristics and the difference in the definition of URTI between different studies.

## 6 Conclusion

The incidence of upper respiratory tract infections among AD patients treated with biological agents is quite variable among included studies across the literature despite the analysis showing low heterogeneity. Nevertheless, the incidence among controls in these RCTs is arguably similar. Biological agents have a bright future for AD patients with good safety profiles. However, more large-scale trials are needed to prove their efficacy and safety, as well as trials that investigate the use of biological agents in different populations, including pediatrics. However, trials should put more control on patients-specific URTI risk factors both in the research and in the clinical decision-making.

## Data Availability

The original contributions presented in the study are included in the article/Supplementary material, further inquiries can be directed to the corresponding author.
