# Supplementary material for: Incidence of upper respiratory tract infections with biological therapies in moderate to severe atopic dermatitis: a systematic review and meta-analysis
Source: Front Med (Lausanne). 2025 Apr 2;12:1550640. doi: 10.3389/fmed.2025.1550640 (PMC12000152; doi:10.3389/fmed.2025.1550640)
Supplement: Supplementary file 1 [file Table_1.docx]

**Supplementary Table 1: Search strategy**

**Pubmed and Medline**

((((("dermatitis, atopic"[MeSH Terms]) OR ("atopic dermatitis"[All Fields])) OR (Eczema, Atopic[MeSH Terms])) OR (Eczema, Atopic)))

AND

(((((((("biologics"[All Fields]) OR ("dupilumab"[All Fields]) OR ("interleukin-4/antagonists and inhibitors receptors"[All Fields]) OR ("interleukins/antagonists and inhibitors"[MeSH Terms]) OR ("tralokinumab"[All Fields]) OR ("interleukin 13 antagonists and inhibitors"[All Fields]) OR ("lebrikizumab"[All Fields])) ) OR ("abrocitinib"[All Fields])) OR ("janus kinase 1/antagonists and inhibitors"[MeSH Terms])) OR ("janus kinase 1 antagonists and inhibitors"[All Fields])) OR ("upadacitinib"[All Fields]))) Filters: Clinical Trial, Randomized Controlled Trial

**Directory of Open Access Journals (DOAJ) (accessed through Saudi Digital Library)**

(Atopic dermatitis)

AND

(Dupilumab OR dupixent OR lebrikizumab OR Tralokinomab OR Upadacitinib OR Abrocitinib OR IL-4 inhibitors OR IL-13 inhibitors OR Janus Kinase 1 antagonist OR JAK1 inhibitor)

AND

RCTs OR Randomized control trials

**ClinicalTrials.gov**

(Atopic dermatitis) OR (Atopic Eczema)

AND

(Dupilumab OR dupixent OR lebrikizumab OR Tralokinomab OR Upadacitinib OR Abrocitinib OR IL-4 inhibitors OR IL-13 inhibitors OR Janus Kinase 1 antagonist OR JAK1 inhibitor)
