# Supplementary material for: Incidence of upper respiratory tract infections with biological therapies in moderate to severe atopic dermatitis: a systematic review and meta-analysis
Source: Front Med (Lausanne). 2025 Apr 2;12:1550640. doi: 10.3389/fmed.2025.1550640 (PMC12000152; doi:10.3389/fmed.2025.1550640)
Supplement: Supplementary file 2 [file Table_2.docx]

**Supplementary Table 2: Risk of bias** **assessment:**

The risk of bias assessment was conducted using ROB-2 for 19 studies. Most studies had a low risk of bias across all domains. However, Simpson 2016 SOLO 1 and SOLO 2 exhibited unclear risk of bias in Selection of the reported result and other bias. Guttman-Yassky 2018 showed a high risk of bias in Selection of the reported result and hence Overall risk of bias. In general, Randomization process, deviations from intended interventions, missing outcome data, and measurement of the outcome showed predominantly low risk. Selection of the reported result demonstrated a mix of low and high risk (1, 5%). Other bias showed a moderate risk in (2, 10%) of studies. The overall risk of bias was primarily low to moderate, with a smaller one study showing high risk.
