# Supplementary material for: Incidence of upper respiratory tract infections with biological therapies in moderate to severe atopic dermatitis: a systematic review and meta-analysis
Source: Front Med (Lausanne). 2025 Apr 2;12:1550640. doi: 10.3389/fmed.2025.1550640 (PMC12000152; doi:10.3389/fmed.2025.1550640)
Supplement: Supplementary file 3 [file Table_3.docx]

| **Study name** | **Participants** | | **Incidence of URTI for treatment group**  **(n; %)** | **Incidence of URTI for control group**  **(n; %)** |
| --- | --- | --- | --- | --- |
|  | **Treatment** | **Control** |  |  |
| Simpson, 2020 ^s1^ | 310 | 77 | (22; 7.1) | (5; 6.5) |
| Worm, 2019 ^s2^ | 338 | 82 | (18; 5.3) | (6; 7.3) |
| Guttman-Yassky, 2018 ^s3^ | 27 | 27 | (4; 14.8) | (4; 14.8) |
| Simpson,2016 ^s4^ | 338 | 82 | (25; 7.3) | (6; 7.3) |
| Guttman-Yassky, 2022 ^s5^ | 301 | 302 | (19; 6.3) | (12; 3.9) |
| Guttman-Yassky, 2022 ^s6^ | 309 | 302 | (17; 5.5) | (12; 3.9) |
| Beck, 2021^s7^ | 46 | 26 | (7; 15.2) | (3; 11.5) |
| Guttman-Yassky, 2021^s8^ | 228 | 52 | (1; 0.4) | (0; 0) |
| Sanofi, 2022 ^s9^ | 82 | 83 | (14; 17.1) | (10; 12) |
| Yassky, 2020 ^s10^ | 41 | 10 | (17; 41.5) | (4; 40) |
| Thaçi, 2017 ^s11^ | 318 | 61 | (23; 7.2) | (11; 18.0) |
| Eli Lilly and Company, 2022 ^s12^ | 282 | 141 | (1; 0.35) | (3; 2.13) |
| Regeneron Pharmaceuticals, 2020 ^s13^ | 97 | 97 | (11; 11.3) | (14; 14.4) |
| Regeneron Pharmaceuticals, 2020 ^s14^ | 155 | 50 | (7; 4.5) | (1; 2) |
| Medlmmune LLC, 2016 ^s15^ | 153 | 51 | (15; 9.8) | (5; 9.8) |
| Simpson, 2019 ^s16^ | 165 | 85 | (32; 19.4) | (15; 17.6) |
| Simpson, 2016 ^s17^ | 447 | 222 | (36; 8.1) | (4; 1.8) |
| Regeneron Pharmaceuticals, 2015 ^s18^ | 27 | 27 | (4; 14.8) | (4; 14;8) |
| ECZTRA 1, 2020 ^s19^ | 602 | 196 | (139; 23.1) | (41; 20.9) |
| ECZTRA 2, 2020 ^s20^ | 592 | 200 | (59; 9.97) | (17; 8.5) |
| ECZTRA 2, 2021^s21^ | 195 | 94 | (19; 9.7) | (4; 4.3) |

**Supplemental table 3.** Incidence of upper respiratory tract infection in the included studies.

Supplemental references:

S1. Study to Evaluate Efficacy and Safety of PF-04965842 in Subjects Aged 12 Years And Older With Moderate to Severe Atopic Dermatitis - Study Results - ClinicalTrials.gov [Internet]. [cited 2023 Apr 12]. Available from: <https://clinicaltrials.gov/ct2/show/results/NCT03349060>

S2. Worm M, Simpson EL, Thaçi D, Bissonnette R, Lacour J-P, Beissert S, et al. Efficacy and Safety of Multiple Dupilumab Dose Regimens After Initial Successful Treatment in Patients With Atopic Dermatitis: A Randomized Clinical Trial. JAMA dermatology. 2020 Feb;156(2):131–43.

S3. Guttman-Yassky E, Bissonnette R, Ungar B, Suárez-Fariñas M, Ardeleanu M, Esaki H, et al. Dupilumab progressively improves systemic and cutaneous abnormalities in patients with atopic dermatitis. J Allergy Clin Immunol. 2019 Jan;143(1):155–72.

S4. Simpson EL, Bieber T, Eckert L, Wu R, Ardeleanu M, Graham NMH, et al. Patient burden of moderate to severe atopic dermatitis (AD): Insights from a phase 2b clinical trial of dupilumab in adults. J Am Acad Dermatol. 2016 Mar;74(3):491–8.

S5. A Study to Evaluate Upadacitinib in Adolescents and Adults With Moderate to Severe Atopic Dermatitis (Measure Up 2) - Full Text View - ClinicalTrials.gov [Internet]. [cited 2023 Apr 12]. Available from: https://clinicaltrials.gov/ct2/show/NCT03607422

S6. A Study to Evaluate Upadacitinib in Adolescents and Adults With Moderate to Severe Atopic Dermatitis (Measure Up 2) - Full Text View - ClinicalTrials.gov [Internet]. [cited 2023 Apr 12]. Available from: <https://clinicaltrials.gov/ct2/show/NCT03607422>

S7. Effect of Dupilumab (Anti-IL4Rα) on the Host-Microbe Interface in Atopic Dermatitis - Full Text View - ClinicalTrials.gov [Internet]. [cited 2022 Nov 6]. Available from: <https://clinicaltrials.gov/ct2/show/NCT03389893>

S8. A Study of Lebrikizumab (LY3650150) in Participants With Moderate-to-Severe Atopic Dermatitis - Full Text View - ClinicalTrials.gov [Internet]. [cited 2022 Nov 6]. Available from: <https://clinicaltrials.gov/ct2/show/NCT03443024>

S9. Evaluation of Dupilumab in Chinese Adult Patients With Moderate to Severe Atopic Dermatitis - Full Text View - ClinicalTrials.gov [Internet]. [cited 2022 Nov 6]. Available from: <https://clinicaltrials.gov/ct2/show/NCT03912259>

S10. A Study to Evaluate ABT-494 (Upadacitinib) in Adults With Moderate to Severe Atopic Dermatitis - Full Text View - ClinicalTrials.gov [Internet]. [cited 2023 Apr 12]. Available from: https://clinicaltrials.gov/ct2/show/NCT02925117

S11. Study of Dupilumab Administered to Adult Patients With Moderate-to-Severe Atopic Dermatitis - Full Text View - ClinicalTrials.gov [Internet]. [cited 2022 Nov 6]. Available from: <https://www.clinicaltrials.gov/ct2/show/NCT01859988>

S12. Evaluation of the Efficacy and Safety of Lebrikizumab (LY3650150) in Moderate to Severe Atopic Dermatitis (ADvocate1) - Full Text View - ClinicalTrials.gov [Internet]. [cited 2022 Nov 6]. Available from: <https://clinicaltrials.gov/ct2/show/NCT04146363>

S13. Study of Dupilumab and Immune Responses in Adults With Atopic Dermatitis (AD) - Full Text View - ClinicalTrials.gov [Internet]. [cited 2022 Nov 6]. Available from: <https://clinicaltrials.gov/ct2/show/NCT02210780>

S14. Efficacy and Safety of REGN3500 Monotherapy and Combination of REGN3500 Plus Dupilumab in Adult Patients With Moderate-to-Severe Atopic Dermatitis - Full Text View - ClinicalTrials.gov [Internet]. [cited 2022 Nov 6]. Available from: <https://clinicaltrials.gov/ct2/show/NCT03736967>

S15. Phase 2 Study to Evaluate the Efficacy and Safety of Tralokinumab in Adults With Atopic Dermatitis - Full Text View - ClinicalTrials.gov [Internet]. [cited 2023 Apr 12]. Available from: https://www.clinicaltrials.gov/ct2/show/NCT02347176

S16. Efficacy and Safety of Dupilumab in Participants ≥12 to <18 Years of Age, With Moderate-to-severe Atopic Dermatitis - Full Text View - ClinicalTrials.gov [Internet]. [cited 2022 Nov 6]. Available from: <https://clinicaltrials.gov/ct2/show/NCT03054428>

S17. Simpson EL, Bieber T, Guttman-Yassky E, Beck LA, Blauvelt A, Cork MJ, et al. Two Phase 3 Trials of Dupilumab versus Placebo in Atopic Dermatitis. N Engl J Med. 2016 Dec;375(24):2335–48.

S18. Study to Determine the Safety and Effectiveness of Dupilumab for Treatment of Atopic Dermatitis (AD) - Full Text View - ClinicalTrials.gov [Internet]. [cited 2022 Nov 6]. Available from: <https://clinicaltrials.gov/ct2/show/NCT01979016>

S19. Tralokinumab Monotherapy for Moderate to Severe Atopic Dermatitis - ECZTRA 1 (ECZema TRAlokinumab Trial no. 1) - Full Text View - ClinicalTrials.gov [Internet]. November 20, 2020. [cited 2022 Dec 24]. Available from: <https://clinicaltrials.gov/ct2/show/study/NCT03131648>

S20. Tralokinumab Monotherapy for Moderate to Severe Atopic Dermatitis - ECZTRA 2 (ECZema TRAlokinumab Trial no. 2) - Full Text View - ClinicalTrials.gov [Internet]. August 24, 2020. [cited 2022 Dec 24]. Available from: <https://www.clinicaltrials.gov/ct2/show/NCT03160885>

S21.
